# Aggregation of cardiovascular risk factors in a cohort of 40-year-olds participating in a population-based health screening program in Sweden

**DOI:** 10.1186/s13690-024-01457-4

**Published:** 2024-11-28

**Authors:** Beata Borgström Bolmsjö, Emelie Stenman, Anton Grundberg, Kristina Sundquist

**Affiliations:** 1 Center for Primary Health Care Research, Department of Clinical Sciences Malmö, Lund University, Malmö, Sweden; 2 University Clinic Primary Care Skåne, Region Skåne, Sweden

**Keywords:** Health intervention program, Targeted health dialogues, Metabolic risk factors, Cardiovascular risk factors, Health behaviors, Primary health care, Primary prevention, Public Health

## Abstract

**Background:**

It is important to identify and evaluate cardiovascular risk factors at an early stage to address them accordingly. Among the younger population, the metabolic syndrome is less common than in older ages. However, each separate metabolic risk factor still has an additive effect on cardiovascular risk factor burden. Non-metabolic risk factors that occur in the younger population include family history, smoking, psychological distress and socioeconomic vulnerability. In 2021 a voluntary health intervention program was introduced in an urban area in Sweden where a cohort of 40-year-olds was invited for cardiovascular risk identification. The aim of this study was to identify how cardiovascular risk factors tend to aggregate in individuals participating in a voluntary health screening program and how the metabolic risk factors associate with non-metabolic cardiovascular risk factors.

**Methods:**

This was a cross-sectional study with 1831 participants. Data from questionnaires and baseline measurements were used to calculate the prevalence of metabolic- (blood pressure, lipids, fasting plasma glucose, BMI, waist-hip ratio) and non-metabolic risk factors (family history of CVD, smoking, psychological distress, socioeconomic vulnerability) for CVD. SCORE2 was calculated according to the algorithm provided by the SCORE2 working group and ESC (European Society of Cardiology) Cardiovascular Risk Collaboration. Associations among each of the metabolic risk factors and non-metabolic risk factors were estimated using logistic regression and presented as odds ratios (ORs) with 95% confidence intervals (CIs).

**Results:**

More than half of the study population had at least one metabolic risk factor, and more than 1/3 was considered to be suffering from psychological distress. Furthermore, obesity or central obesity demonstrated individual associations with all of the non-metabolic risk factors in the study; smoking (1.49; 1.32–2.63), family history of CVD (1.41; 1.14–1.73), socioeconomic vulnerability (1.60; 1.24–2.07), and psychological distress (1.40; 1.14–1.72). According to SCORE2 25% of the men were at moderate risk (2.5–7.5%) of developing a cardiovascular event within 5–10 years, but only 2% of the women.

**Conclusions:**

Obesity/central obesity should be a prioritized target in health screening programs. The non-metabolic risk factors, socioeconomic vulnerability, and psychological distress should not be ignored and addressed with adequate guidance to create health equity.

**Table:** 

**Text box 1. Contributions to the literature**
• In a community-based health intervention program for 40-year-olds in Sweden, more than half had at least one metabolic risk factor, where obesity was the most common. In addition, over one third suffered from psychological distress.
• Obesity/abdominal obesity demonstrated individual associations with all of the non-metabolic risk factors (smoking, family history of CVD, socioeconomic vulnerability, and psychological distress)
• Having no non-metabolic risk factor was more common in the group without any metabolic risk factor.
• To gain further impact on cardiovascular risk, intervention programs could benefit from a stronger focus on certain patient groups, for example those with obesity/central obesity.

## Background

For most of the 20th century, cardiovascular disease (CVD) was identified as the major cause of morbidity and mortality in the developed world. CVD, and especially coronary artery disease, is still the major cause of premature death in Europe and the leading somatic cause of productivity loss [1, 2]. Hence, it is important to identify and evaluate CVD risk factors in adults to address and treat them accordingly. Such public health initiatives should preferably be performed in primary health care settings, where most patients are treated, as well as in socioeconomically and diverse populations. It is also important to associate such initiatives with research studies that can contribute to a novel knowledge base for improved health in the whole population as well as in vulnerable population groups. This will be done in the present study where we expect that the research findings will be useful in most parts of Sweden that have implemented population-based primary preventive interventions as well as in other countries similar to Sweden.

Aggregation of metabolic risk factors increases the cardiovascular risk further, which then becomes greater than the risk associated with the individual components [3]. As cardiovascular risk factors tend to cluster, they also share receptivity to lifestyle modifications. For example, lifestyle interventions that lead to weight loss are beneficial for hyperglycemia, hyperlipidemia and hypertension [4]. Hence, a change to healthier lifestyle habits can reduce the overall cardiovascular risk by reducing several metabolic risk factors [5].

WHO defined metabolic syndrome in 1998 as a cluster of several known cardiovascular risk factors, including insulin resistance, obesity, atherogenic dyslipidemia and hypertension [6]. Regarding cardiovascular risk, it is important to emphasize that the definition of metabolic syndrome does not include sex, smoking, family history or age, which also constitute important cardiovascular risk factors [7].

An alternative approach to assessing aggregated cardiovascular disease (CVD) risk factors is the use of risk estimators. In 2021, the European Society of Cardiology (ESC) introduced SCORE2, designed to identify individuals with varying levels of CVD risk, including younger populations. SCORE2 estimates risk of both fatal and non-fatal CVD events, incorporating risk factors such as age, sex, blood pressure, smoking, and non-HDL cholesterol [8].

A meta-analysis including 19 eligible studies showed that depression is prospectively associated with a significant increase in the risk of myocardial infarction (MI) and coronary death [9].

Socioeconomic burden as well as psychosocial factors that influence health also play a significant role in the risk of CVD [10], and studies show that psychosocial conditions, such as self-reported chronic stress as well as experienced loneliness and unemployment, are independent risk factors for CVD [11–13]. It has been suggested that socioeconomic eligibility criteria may be considered as additional eligibility criteria for CVD risk screening, as they improve detection of CVD risk [14].

As a result of the high disease burden, several interventions have been conducted targeting CVD risk factors and the increased prevalence of metabolic syndrome and diabetes mellitus type 2 (DM2). These have been shown to be highly cost-effective and ultimately cost-saving (WHO 2018). The Swedish intervention program, Targeted Health Dialogues (THD), is a method to explore risk factors for CVD and DM2 in the population to offer support and guidance for lifestyle change. THDs have been offered since the 1980s in different parts of Sweden [15]. THDs have been shown to be a cost-effective intervention [15] and have also been demonstrated with moderate levels of evidence to reduce the overall mortality rate as well as the mortality for cardiovascular disease [16, 17]. The population in the area where THDs are conducted is invited by age groups to participate in THDs at their primary health care center. THDs differ from ordinary health checks in that they specifically target CVD risk factors and are not attached to any costs for the individual. The THDs are person-centered and the dialogues, using a motivational interviewing technique, originate from the person’s own best interests and conditions thus resulting in a combination of health promotion and disease prevention measures.

Primary care in Sweden is overburdened, with limited resources, and implementing health screening programs conducted at primary health care centers has therefore been questioned [18]. According to Geoffrey Rose’s population approach, even small reductions in risk factors across the population can substantially reduce disease prevalence. This can be compared to a high-risk approach, which focuses on individuals at the highest risk, allowing precise, personalized care but impacting only on a small portion of the population [19]. Balancing these approaches—combining targeted interventions for high-risk individuals with broad public health strategies, such as the THDs in Sweden—may be a strategy to enhance the overall public health impact. The THDs were introduced in our county, the third most populous region in Sweden, in 2021 and the THD research cohort has grown over time. In a previously published study of ours, we showed that only 31.6% of the participants were sufficiently physically active and that 60% had poor eating habits. Insufficient physical activity was also significantly associated with poor eating habits. When comparing those who had three or more unhealthy behaviors with those who had no unhealthy behaviors, we found that they had a higher prevalence of several metabolic risk factors [20].

The aim of the present study was to explore the occurrence of metabolic risk factors in a cohort of generally healthy 40-year-olds participating in THDs. Furthermore, we wanted to explore the associations with non-metabolic risk factors such as heredity, smoking, socioeconomic burden and psychological distress, which all constitute factors that may imply an additional risk of future CVD.

## Methods

### Setting and study population

The present study was based on data derived from the baseline measurements of 40-year-old participants in the research project of THDs conducted in Skåne county. Skåne is the southernmost county of Sweden with about 1.4 million inhabitants living in both rural and metropolitan areas. More than 20% of Skåne’s inhabitants are born outside Sweden, with the majority originating from Syria, Iraq and Poland [21].

Inhabitants at participating PHCCs (prrimary healthcare centers) in Skåne, who turned 40 years old in 2021 or 2022, were invited to participate in the THDs. They were also informed about, and invited to, participate in the research project of the THDs. Being part of the research project meant that the research group could take part of the data collection in the THDs and follow the participants’ morbidity in medical records for a 10-year follow-up time. It was possible to participate in the THDs without participating in the research project. Written informed consent was a prerequisite for taking part in the research project. Ethical approval was obtained from the Swedish Ethical Review Authority (registration number 2020–02689 with later amendments). The study is registered at ClinicalTrials.gov, identifier: NCT04912739.

### The targeted health dialogues (THD)

THDs have been described in detail previously [22]. An invitation to a THD was sent out by letter to 40-year-olds registered at each healthcare center. Before the health dialogue, the participants filled in a questionnaire about background factors, such as country of origin, education, current life situation as well as psychosocial health, self-rated health, previous diseases, chronic diseases, family history of diseases, and health behaviors (tobacco, alcohol, diet, physical activity). At the first visit to the healthcare center, fasting blood tests were drawn for total cholesterol, low-density lipoprotein (LDL)-cholesterol, high-density lipoprotein (HDL)-cholesterol, and fasting plasma glucose. Body mass index (BMI), waist–hip ratio and blood pressure were also measured before the health dialogue. Blood pressure was measured in a sitting position after resting for five minutes. It was measured in both arms at least two times with one minute in between each measurement. The mean value was then registered. Around a week later, the participants went through a THD led by a specially trained health dialogue coach. At the THD, the health dialogue coach and participant together went through all the results from the questionnaire and measurements. Strategies regarding optimized health behaviors were discussed by use of motivational interviewing.

### Data collection

The data in the THDs were stored in a quality register within Region Skåne. From there, pseudonymized data for those who had consented to take part in the research project were transferred to a research database. Data went through a monitoring process to ensure internal validity.

### Variables regarding metabolic risk factors

Five metabolic variables were included in the analyses: blood pressure, lipids, fasting plasma glucose, BMI, and waist-hip ratio. The variables were composed and dichotomized as described below.

High blood pressure was defined as systolic blood pressure ≥ 140 mmHg or diastolic blood pressure ≥ 90 mmHg according to European guidelines [23]. In this study, we grouped patients with blood pressure ≥ 140/90 or/and those who reported antihypertensive treatment in the composed variable High blood pressure.

The composed variable high cholesterol was defined according to international guidelines [24] as having fasting cholesterol ≥ 7.5 mmol/L or LDL-cholesterol ≥ 5 mmol/L or having cholesterol treatment.

High fasting plasma glucose level was defined as ≥ 6.1 mmol/L, which is the limit for impaired glucose tolerance according to the WHO. A suspicion of diabetes was defined at ≥ 7 mmol/L [25]. The composed variable High blood glucose included patients with F-plasma glucose ≥ 6.1 mmol/L or DM2 diagnosis.

Obesity was defined as BMI ≥ 30 kg/m^2^ [26]. High waist–hip ratio was defined as ≥ 0.85 for women and ≥ 0.90 for men according to the WHO definition of abdominal obesity in the metabolic syndrome [27]. Obesity and waist-hip ratio were grouped together to a composed metabolic risk factor defined as BMI ≥ 30 or WHR ≥ 0.9 (m) /0.85 (w).

SCORE2 was calculated according to the algorithm provided by the SCORE2 working group and the ESC Cardiovascular Risk Collaboration [8].

### Variables regarding non-metabolic cardiovascular risk factors

While metabolic risk factors may be less prominent in younger populations, it is also important to consider other non-metabolic risk factors when assessing overall cardiovascular risk. Non-metabolic risk factors considered in this study were: (1) family history of CVD, (2) smoking, (3) psychological distress and (4) socioeconomic vulnerability.

Family history of CVD was defined as having answered yes to the question of having any mother/father/sibling or child with CVD.

Smoking was defined as answering yes to the question “do you smoke cigarettes daily”.

The variable psychological distress is a composed measure of the following questions: Have you during the last 12 months had any of the following symptoms: sleeping problems, anxiety, depressive symptoms, or fatigue (each of these generated one point) and the level of stress the participant indicated on a stress scale (generated maximum three points). If the answers summed up to five points or more, the participant was considered to be suffering from psychological distress,

Socioeconomic vulnerability was defined as a composed measure of several answers to questions about the participants’ life situation. The following situations each generated one point:

-If the answer was “no” to the question: *Are you married or are you living with your partner?*

-If the answer was “no” or “only to some extent” to the question: *Do you have someone you can share your innermost feelings with and confide in?*

-If the answer was “yes” to the questions: *Are you unemployed/on permanent sick leave/retired?* Or *Do you expect to become unemployed during the coming year?*

-If the answer was “yes” or “partly yes” to the question: *Are your finances a problem for you?*

If the answers summed up to two points or more, the participant was considered as being socioeconomically vulnerable.

### Statistical analysis

Participant characteristics are presented as categories with numbers and percentages. Pearson’s Chi-squared tests were used to test for potential differences in characteristics between men and women. To assess associations among each of the metabolic risk factors and the non-metabolic risk factors, odds ratios (ORs) with 95% confidence intervals (CI) were estimated using logistic regression. The associations between the non-metabolic cardiovascular risk factors and the group with no metabolic risk factors as well as one or more metabolic risk factors as outcomes were estimated as ORs using logistic regression. The ORs for each logistic regression analysis were also estimated after adjusting for potential confounders including sex, level of education, and country of origin. The chosen level of statistical significance was 0.05. All statistical analyses were done in R version 4.2.1 [28].

## Results

### Population characteristics

The 99 PHCCs in the study invited 8 479 40-year-olds to a THD. Of these, 3985 persons accepted the invitation and 2937 completed their THD during the period of data collection for this study. A total of 1831 participants (62.3%) consented to be part of this research project, and hence constitute the study population. All participants were at the age of 40 years and there were slightly more women than men (55.2% vs. 44.8%). Only 5.8% had an educational level of less than nine years and the majority of the participants were born in Sweden (68.4%) (Table 1).

Regarding metabolic risk factors, less than half of the population were of normal weight (45.5% with BMI < 25) but 59.5% had normal waist to hip ratio (WHR) (data not shown). For the composed measure regarding obesity that is included in metabolic syndrome i.e., BMI ≥ 30 or WHR ≥ 0.9 (male)/0.85 (female), 44.7% of the study population met these criteria A significantly larger prevalence of obesity and high WHR was observed in men compared to women (57% vs. 34.8%) (Table 1).

Around 20% of the population had hypertension and/or antihypertensive treatment whereas around 6% had hyperlipidemia (cholesterol ≥ 7.5 or LDL ≥ 5) or cholesterol treatment. Almost 10% of the population had fasting glucose levels corresponding to DM2 or prediabetes (fasting plasma glucose > 6) (Table 1).

Less than half of the participants did not have any metabolic risk factor. Around one third had one metabolic risk factor and around 20% had two or more metabolic risk factors. An obvious difference also emerged between genders: 31.5% of men had no metabolic risk factors, compared to over half of the women who were free of any risk factors.

Regarding non-metabolic cardiovascular risk factors, family history of CVD was reported in 37.7%, and 8.0% reported smoking. Psychological distress was reported by 34.6% and socioeconomic vulnerability was reported by 17.9% of the participants (Table 1). The gender difference was evident, with a higher proportion of men having a family history of cardiovascular disease, while women were more likely to experience psychological distress.

As reported previously [20], the baseline characteristics differed slightly from the age matched population in that there were more women in our study, i.e., 55.2% vs. 49.7%. The proportion having education > 12 years was higher, i.e., 61.2% vs. 55.1% and there was also a higher proportion of Swedish born participants in the study population compared to the age matched population in Scania, i.e., 68.2% vs. 61.2% [20].

**Table 1:** Population characteristics stratified by sex for the participants in Targeted Health Dialogues in Scania, Sweden, Jan 2021- June 2022. Data is presented as n (%)

	Characteristics	Total *n* = 1831	Men *n* = 821	Women *n* = 1010	*p*-value
**Demographic data**	Sex				< 0.001
	Men	821 (44.8)	-	-	
	Women	1010 (55.2)	-	-	
	Level of education				< 0.001
	≤ 9 years	107 (5.8)	59 (7.2)	48 (4.8)	
	10–12 years	602 (32.9)	330 (40.2)	272 (26.9)	
	> 12 years	1120 (61.2)	431 (52.5)	689 (68.2)	
	Missing	2 (0.1)	1 (0.1)	1 (0.1)	
	Country of origin				0.86
	Sweden	1252 (68.4)	561 (68.3)	691 (68.4)	
	Other European country	270 (14.7)	118 (14.4)	152 (15.0)	
	Non-European country	309 (16.9)	142 (17.3)	167 (16.5)	
**Metabolic risk factors**	BMI ≥ 30 or WHR ≥ 0.9 (m)/0.85 (f)				< 0.001
	Yes	819 (44.7)	468 (57.0)	351 (34.8)	
	No	992 (54.2)	343 (41.8)	649 (64.3)	
	Missing	20 (1.1)	10 (1.2)	10 (1.0)	
	Blood pressure ≥ 140/90 or antihypertensive treatment				< 0.001
	Yes	361 (19.7)	226 (27.5)	135 (13.4)	
	No	1466 (80.1)	593 (72.2)	873 (86.4)	
	Missing	4 (0.2)	2 (0.2)	2 (0.2)	
	Cholesterol ≥ 7.5 or LDL ≥ 5 or cholesterol treatment				< 0.001
	Yes	107 (5.8)	77 (9.4)	30 (3.0)	
	No	1710 (93.4)	738 (89.9)	972 (96.2)	
	Missing	14 (0.8)	6 (0.7)	8 (0.8)	
	F-plasma glucose > 6 or DMII diagnosis				< 0.001
	Yes	174 (9.5)	111 (13.5)	63 (6.2)	
	No	1641 (89.6)	705 (85.9)	936 (92.7)	
	Missing	16 (0.9)	5 (0.6)	11 (1.1)	
	Numbers of metabolic risk factors				< 0.001
	0	821 (44.8)	259 (31.5)	562 (55.6)	
	1	585 (31.9)	288 (35.1)	297 (29.4)	
	2	298 (16.3)	191 (23.3)	107 (10.6)	
	3	74 (4.0)	55 (6.7)	19 (1.9)	
	4	11 (0.6)	10 (1.2)	1 (0.1)	
	Missing	42 (2.3)	18 (2.2)	24 (2.4)	
**Non-metabolic risk factors**	Daily smoking				0.66
	Yes	146 (8.0)	68 (8.3)	78 (7.7)	
	No	1683 (91.9)	752 (91.6)	931 (92.2)	
	Missing	2 (0.1)	1 (0.1)	1 (0.1)	
	Family history of CVD				< 0.001
	Yes	691 (37.7)	267 (32.5)	424 (42.0)	
	No	962 (52.5)	473 (57.6)	489 (48.4)	
	Don’t know	178 (9.7)	81 (9.9)	97 (9.6)	
	Psychological distress				< 0.001
	Yes	634 (34.6)	232 (28.3)	402 (39.8)	
	No	1197 (65.4)	589 (71.7)	608 (60.2)	
	Socioeconomic vulnerability				0.52
	Yes	327 (17.9)	152 (18.5)	175 (17.3)	
	No	1500 (81.9)	668 (81.4)	832 (82.4)	
	Missing	4 (0.2)	1 (0.1)	3 (0.3)	

### Associations between metabolic risk factors and non-metabolic risk factors

The only metabolic risk factor that was associated with each of the non-metabolic risk factors was obesity/central obesity, when adjusted for sex, level of education and country of origin. High blood pressure was associated with CVD family history, and dyslipidemia was associated with CVD family history and psychological distress (Table 2).

**Table 2:** Associations of metabolic risk factors with non-metabolic risk factors for the participants in Targeted Health Dialogues in Scania, Sweden, Jan 2021–June 2022. Data are shown as OR (95% CI)

	High glucose		High blood pressure		High BMI or WHR		High cholesterol	
	F-plasma glucose > 6 or DMII diagnosis		Blood pressure ≥ 140/90 or antihypertensive treatment		BMI ≥ 30 or WHR ≥ 0.9 (m)/0.85 (f)		Cholesterol ≥ 7.5 or LDL ≥ 5 or cholesterol treatment	
	Univariable	Adjusted^a^	Univariable	Adjusted^a^	Univariable	Adjusted^a^	Univariable	Adjusted^a^
Psychological distress	0.79 (0.56–1.10)	0.90 (0.63–1.26)	0.96 (0.75–1.22)	1.07 (0.83–1.37)	1.19 (0.98–1.44)	**1.40 (1.14–1.72)**	**1.65 (1.11–2.44)**	**2.05 (1.36–3.08)**
Socioeconomic vulnerability	**1.58 (1.09–2.27)**	1.44 (0.98–2.08)	0.96 (0.71–1.30)	0.95 (0.69–1.30)	**1.76 (1.38–2.25)**	**1.60 (1.24–2.07)**	**1.59 (0.99–2.47)**	1.44 (0.89–2.28)
Smoking	1.36 (0.79–2.24)	1.07 (0.60–1.80)	1.05 (0.68–1.58)	1.03 (0.65–1.58)	**1.86 (1.32–2.63)**	**1.49 (1.03–2.15)**	1.19 (0.57–2.23)	0.92 (0.43–1.81)
CVD family history	0.91 (0.65–1.27)	1.01 (0.71–1.41)	1.22 (0.96–1.55)	**1.41 (1.10–1.81)**	**1.23 (1.01–1.50)**	**1.41 (1.14–1.73)**	**1.69 (1.12–2.56)**	**1.93 (1.27–2.95)**

^a^ Adjusted for sex, level of education and country of origin.

Bold text indicates p-value < 0.05

WHR: Waist to hip ratio, BMI: Body mass index, CVD: Cardiovascular disease

As shown in Table 3 individuals with one or more metabolic risk factors had higher odds ratios for having each of the non-metabolic risk factors, aside from smoking, when adjusted for sex, level of education and country of origin.

**Table 3:** Associations of individuals with ≥ 1 metabolic risk factors with non-metabolic risk factors for the participants in Targeted Health Dialogues in Scania, Sweden, Jan 2021–June 2022. Data are shown as OR (95% CI)

	≥1 metabolic risk factor (ref. 0 metabolic risk factors)	
	Univariable	Adjusted^a^
Psychological distress	1.09 (0.90–1.33)	**1.29 (1.05–1.59)**
Socioeconomic vulnerability	**1.56 (1.22–2.01)**	**1.42 (1.09–1.85)**
Smoking	**1.55 (1.10–2.22)**	1.25 (0.86–1.84)
CVD family history	**1.24 (1.02–1.52)**	**1.46 (1.18–1.80)**

^a^ Adjusted for sex, level of education and country of origin

Bold text indicates p-value < 0.05

WHR: Waist to hip ratio, BMI: Body mass index, CVD: Cardiovascular disease

**Fig. 1:**
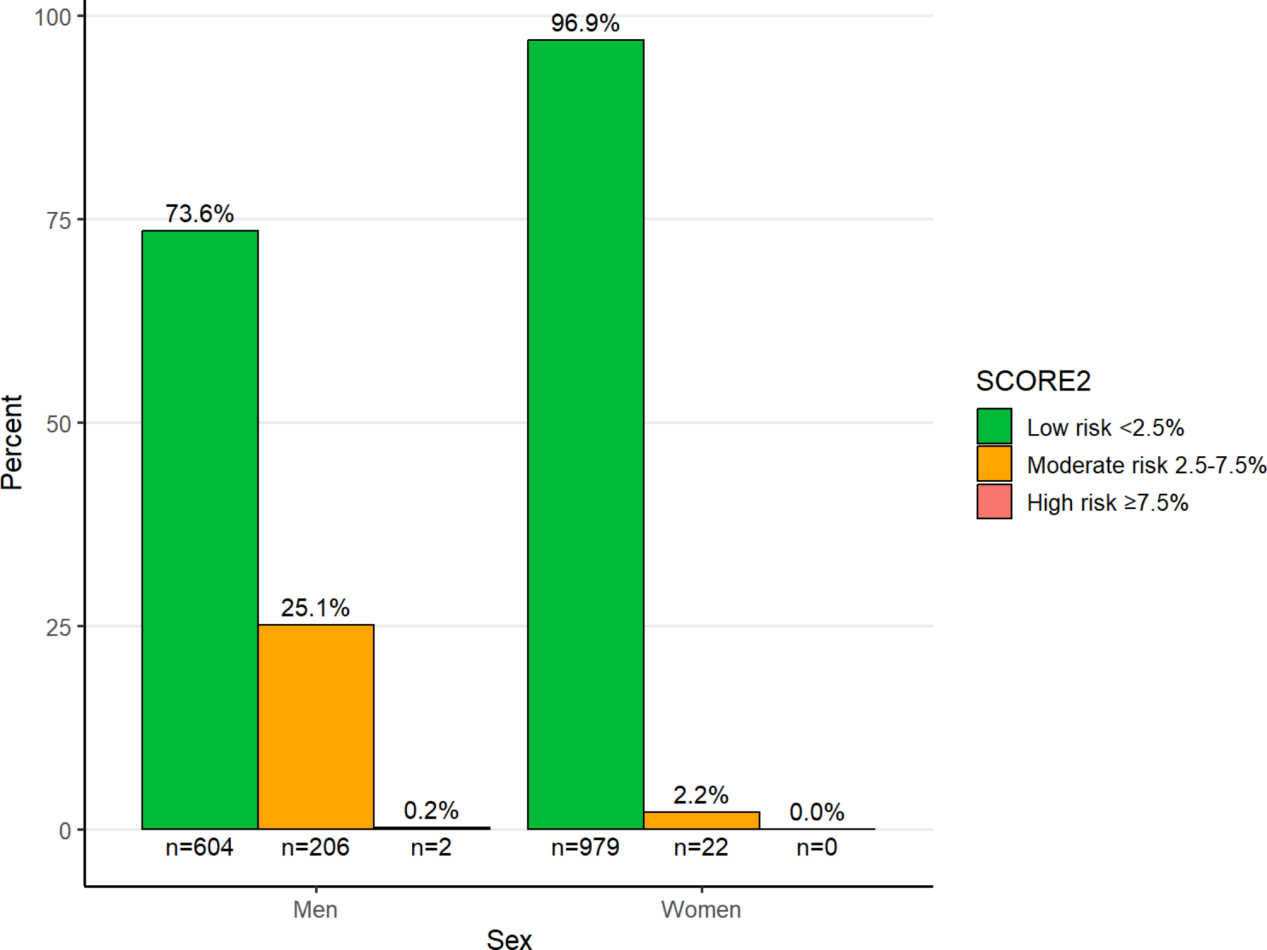
Distribution of SCORE2 in men and women participating in Targeted HealtDialogues in Scania, Sweden, Jan 2021- June 2022

Figure 1 shows the proportions of the population in the different SCORE2 categories of CVD risk based on gender. Almost all women were in the green category i.e. having a low 10-year risk of CVD event (fatal or non-fatal), while one fourth of the men were in the orange category (moderate risk) with between 2.5 and 7.5% 10-year risk of a CVD-event.

## Discussion

Our study shows that more than half of this relatively young population had at least one metabolic risk factor with obesity/central obesity being the most common and more than 1/3 were considered to be suffering from psychological distress. The gender differences were obvious with a higher extent of metabolic risk factors and CVD family history among men and more psychological distress among women. Furthermore, the metabolic risk factors tended to aggregate with the non-metabolic risk factors; psychological distress, socioeconomic vulnerability, CVD family history and smoking. Having at least one metabolic risk factor was associated with all the non-metabolic risk factors in the study except for smoking, when adjusted for sex, level of education and country of origin. Obesity/central obesity was associated with each of the non-metabolic risk factors.

Our results show a better CVD risk profile compared to a cross-sectional study from China where 65.3% of the population ≥ 18 years had at least one CVD risk factor. This difference can likely be attributed to the prevalence of smoking, which is far more prevalent in China compared to Sweden [29].

The benefits of population-based health intervention programs may vary. A Cochrane review from 2011 states that interventions using counseling aiming for behavioral changes have limited use in general populations and do not reduce cardiovascular mortality in those populations although they may be effective in reducing mortality in high-risk hypertensive and diabetic populations [30]. However, the approach of the Swedish population-based THDs seems to be beneficial and the THDs have shown positive effects on CVD [17]. As we show, almost half of the population did not have any metabolic risk factor and may not be a cost-effective target for health intervention. On the other hand, more than half of the population had at least one modifiable metabolic risk factor that could be reduced by a healthier lifestyle [31] and perhaps could benefit from a combination of preventive medications [32]. In a previous study of ours, based on the same cohort, we found that many participants were insufficiently physically active and that they often had poor eating habits. In addition, insufficient physical activity was significantly associated with poor eating habits [20]. The present study shows that the metabolic risk factors tend to aggregate in the individuals, and there should therefore be considerable and multiple health benefits with targeted lifestyle recommendations focusing on increasing physical activity and improving unhealthy dietary habits.

Obesity/central obesity is a common modifiable risk factor, and the most prevalent metabolic risk factor in this study, also linked to all non-metabolic risk factors. Considering the relatively young age of the study population, it is possible that obesity is the first symptom of increased cardiovascular risk and that other metabolic risk factors will occur in the future [4]. A study from the U.S. has shown that interventions targeted at obesity in women, people with lower education, and smokers may result in a significant reduction of multimorbidity. However, they concluded that interventions may need to focus on people prior to mid-life to have the greatest effect [33], which supports the decision to include ages 40 years in the THDs. A large American study showed a strong association between obesity and psychological distress among young adults, with particularly pronounced effects on non-Hispanic White individuals and women [34]. These findings are in line with ours and important for future health policies. Addressing both psychological distress and obesity through lifestyle interventions—such as dietary advice, physical activity, and structured exercise programs—has shown promising results regarding both mental health and obesity outcomes and provides an excellent foundation for preventive CVD programs [35]. Targeting obesity could indeed yield substantial benefits, but focusing solely on primary care services for prevention may be less effective than also engaging policymakers and society at large. A study on the concept of ‘time needed to treat’ indicates that adhering to NICE guidelines for lifestyle counseling would require a substantial increase in primary healthcare staffing, potentially leading to less priority to other areas of health care [36]. Other types of intervention may also be needed. For example, by involving policy makers, governments may add to the effects of primary care interventions by enabling legislation to subsidize healthy foods, and create programs that promote physical activity, which might add to the capacity of primary care to impact health on a large scale.

Elevated blood pressure exhibited an association with a family history of cardiovascular disease (CVD). This observation holds significance, particularly within the context of this study involving a younger population. Family history tends to contribute to the early onset of hypertension, thus emphasizing the need for consideration even in younger age groups. Despite being a non-modifiable risk factor, the inclusion of family history in a screening program remains crucial. Identifying individuals with such familial predispositions is essential for offering targeted lifestyle advice, thereby optimizing health outcomes. However, aiming at this fairly young population of 40-year olds may still not be sufficient according to other studies, pointing at the need for preventive management of CVD already in adolescence and early adulthood [37].

The importance of reaching out to patients with socioeconomic vulnerability and psychological distress should not be underestimated [38, 39], as we show the association between metabolic risk factors and socioeconomic vulnerability and psychological distress.

According to SCORE2 risk assessments, one fourth of the men in our study were at moderate risk of experiencing a cardiovascular event within the next 10 years, compared to only 2% of the women. Previous research suggests that SCORE2 may both underestimate and overestimate cardiovascular risk, as it does not consider non-metabolic risk factors like socioeconomic status or ethnicity [40, 41]. Our study found indeed that the number of metabolic risk factors was associated with the included non-metabolic risk factors, also when accounting for gender. Thus, a risk estimation by should preferably take such risk factors into account as well. The THDs were implemented according to a well-established protocol [42] with educated study personnel conducting structured objective measurements of baseline parameters including BMI, WHR and other metabolic risk factors. The THD questionnaire and measurements cover a broad range of cardiovascular risk factors which allows a thorough risk assessment in these relatively young individuals. These measures aimed at reaching as many people as possible, both rural and urban, and also in segregated areas, as well as in different socioeconomic levels of the society. When the participating population was compared to the age-matched population in Skåne, there was a small but significant difference with higher educational level, more women, and more native-born Swedes in the THD group compared to the population in Skåne [20]. This creates a participation bias, which is common in many intervention programs where the interventions do not reach the most vulnerable inhabitants [43]. Given that, it is possible that the prevalence of cardiovascular risk factors in the present study is even underestimated.

As socioeconomic vulnerability and psychological distress were self-reported, they may be influenced by self-reporting bias. However, it is the individual’s perspective on these issues that we find important for intrinsic motivation and therefore value higher than measures from registers. The questionnaires on psychological distress and socioeconomic vulnerability were specifically designed for use in the THDs. The primary intention behind these questionnaires was to serve as a starting point for discussion with the health dialogue coach, rather than being a research tool. This may limit the reliability and validity of the questionnaires for research studies. To improve validity, we therefore chose to include multiple items for each definition, allowing us to more comprehensively capture different aspects of the concepts of psychological distress and socioeconomic vulnerability.

The limited prevalence of daily smokers within the study population necessitates a cautious interpretation of statistical comparisons related to this risk factor. A more extensive cohort may have yielded divergent results. Another limitation is that the variable family history did not consider age of the affected family member although premature CVD is more likely to have genetic causes.

Our results showed that a large proportion of 40-year-olds that participate in a health screening program targeting cardiovascular risk suffer from at least one metabolic risk factor and that these risk factors are associated with additional cardiovascular risk. Obesity was associated with all other non-metabolic risk factors and may be the first sign of increased cardiovascular risk, which should be taken into consideration in this population. Risk assessed by SCORE2 showed that men are at a much higher risk compared to women, but a larger proportion of women suffer from psychological distress than the men, which is not accounted for in the risk calculators. The non-metabolic risk factors; psychological distress and socioeconomic vulnerability should not be ignored and must be reached out to and faced with adequate guidance to create health equity.

## Data Availability

Raw data are not publicly available to preserve individuals’ privacy under the European General Data Protection Regulation. The datasets used and/or analysed during the current study are available from the corresponding author on reasonable request.

## Abbreviations

BMI: Body mass index
CVD: Cardiovascular disease
DM2: Diabetes Mellitus type 2
HDL: High-density lipoprotein
LDL: Low-density lipoprotein
PHCC: Primary Healthcare Center
THDs: Targeted Health Dialogues
WHR: Waist to hip ratio
WHO: World Health organization
